# Supplementary material for: The NMD Pathway Regulates GABARAPL1 mRNA during the EMT
Source: Biomedicines. 2021 Sep 23;9(10):1302. doi: 10.3390/biomedicines9101302 (PMC8533616; doi:10.3390/biomedicines9101302)
Supplement: Supplementary file 1 [file biomedicines-09-01302-s001.zip › biomedicines-1341755-supplementary.pdf]

Supplementary files

The NMD pathway differentially regulates ATG8 mRNAs during EMT

Timothée Baudu [1], Chloé Parratte [1], Valérie Perez [1], Anne Peigney [1,5], Paul Peixoto [1,5], Eric Hervouet [1,4,5], Marie Ancion [2], Michael Herfs [2], Stefania Millevoi [3], Annick Fraichard [1], Michael Guittaut [1,4] and Aurélie Baguet [1,4,*]

[1] Univ. Bourgogne Franche-Comté, INSERM, EFS BFC, UMR1098, RIGHT Institute, Interactions Hôte-Greffon-Tumeur/Ingénierie Cellulaire et Génique, F-25000 Besançon, France.
[2] Laboratory of Experimental Pathology, GIGA-Cancer, University of Liege, B-4000 Liege, Belgium.
[3] Cancer Research Centre of Toulouse, INSERM UMR 1037, Université Toulouse III - Paul Sabatier, 31330 Toulouse, France.
[4] DImaCell platform, Univ. Bourgogne Franche-Comté, F-25000 Besançon, France.
[5] EPIGENEXP platform, Univ. Bourgogne Franche-Comté, F-25000 Besançon, France.

* Correspondence: aurelie.baguet@univ-fcomte.fr

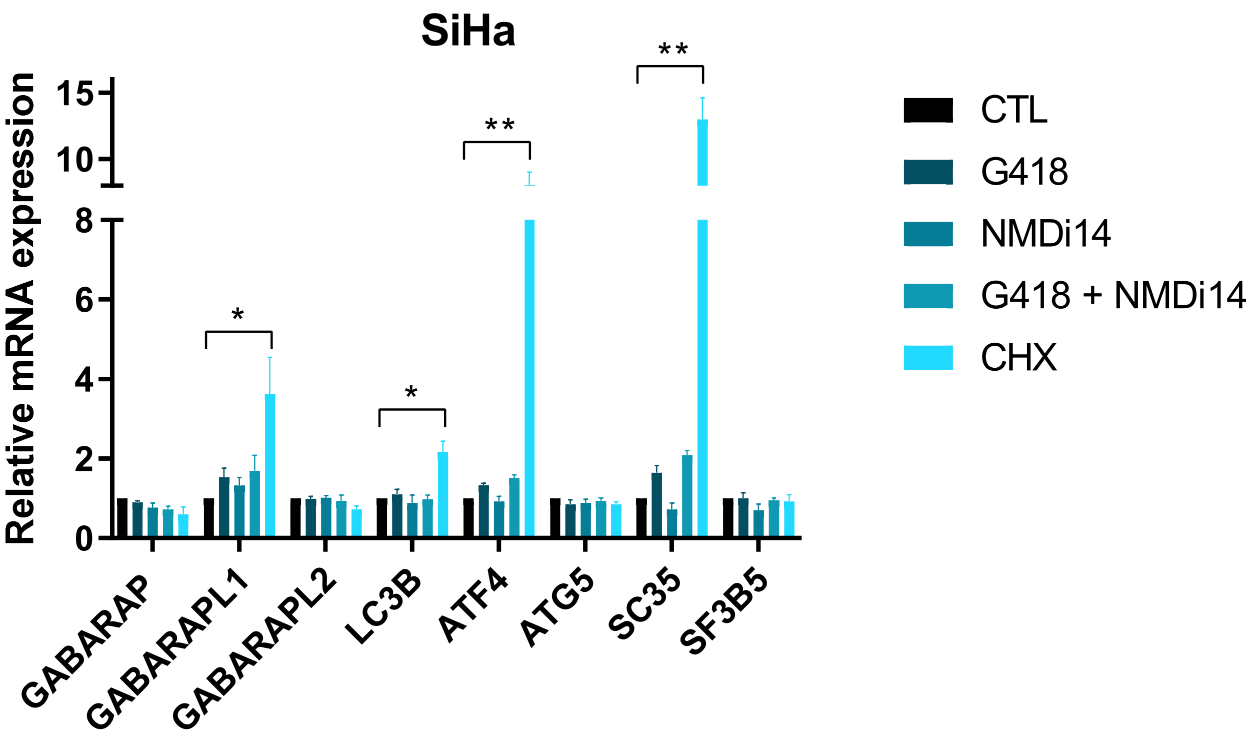

Figure S1. Regulation of the ATG8 family mRNA expression upon chemical inhibition of the NMD. *SiHa cells have been treated with G418 (400 µg/ml) or NMDi14 (50 µM) for 24 h, or with CHX (100 µg/ml) for 5 h, and the expression levels of ATG8 mRNAs have been analyzed by RT-qPCR. The values were calculated using the ΔΔCT method and were relative to H3B2 levels and expressed as fold change. Data are represented as ±S.E.M of three independent experiments. P-values were calculated using Student's t test.*

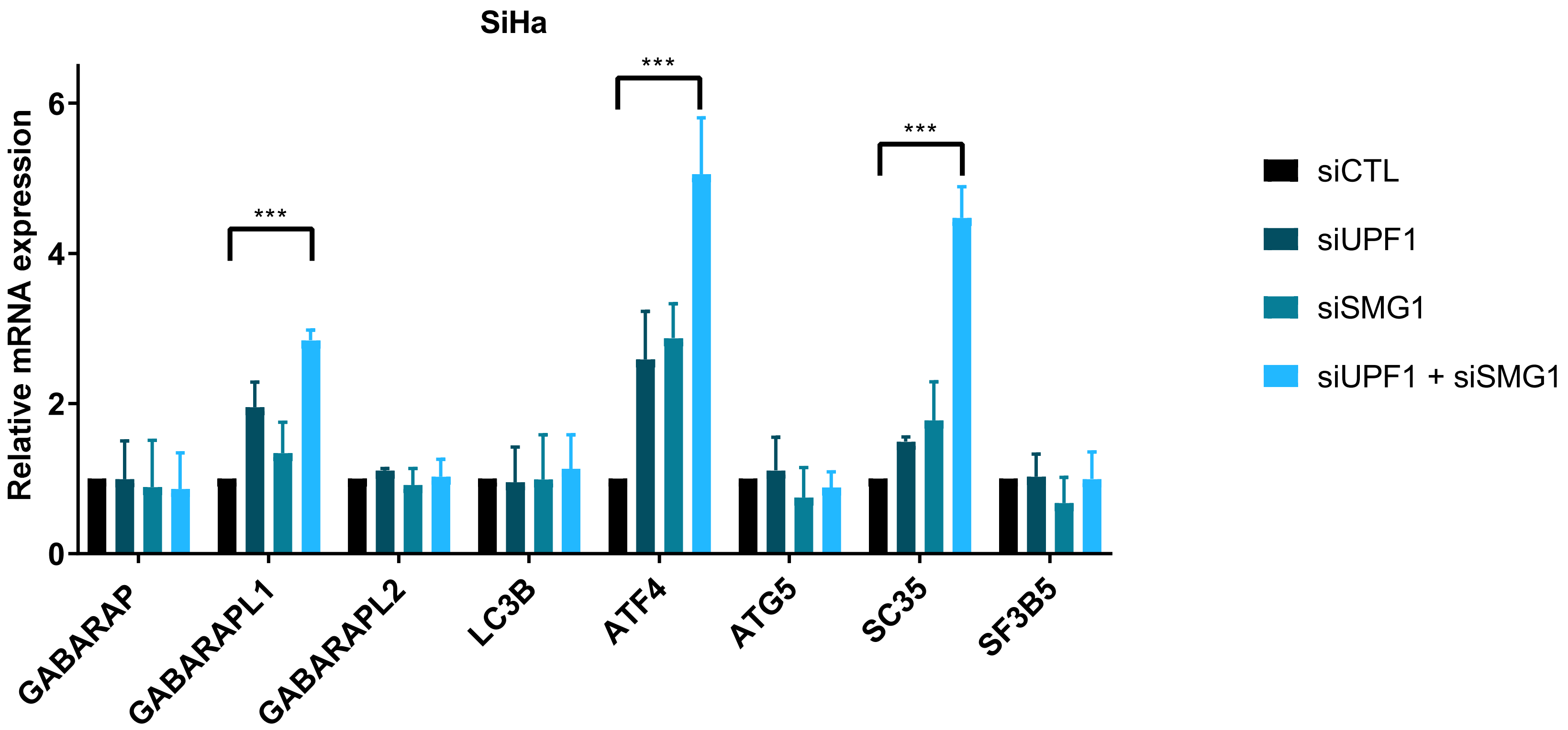

Figure S2. Regulation of the ATG8 family mRNAs expression upon siRNA-mediated inhibition of the NMD. *SiHa cells have been transfected with siRNA (20 pmol/well) targeting NMD transcripts UPF1 or SMG1. Quantification by RT-qPCR of ATG8 transcripts expression levels. The values were calculated using the ΔΔCT method and were normalized to H3B2 levels and expressed as fold changes. Data are represented as ± S.E.M of three independent experiments. P-values were calculated using a Student's t test.*

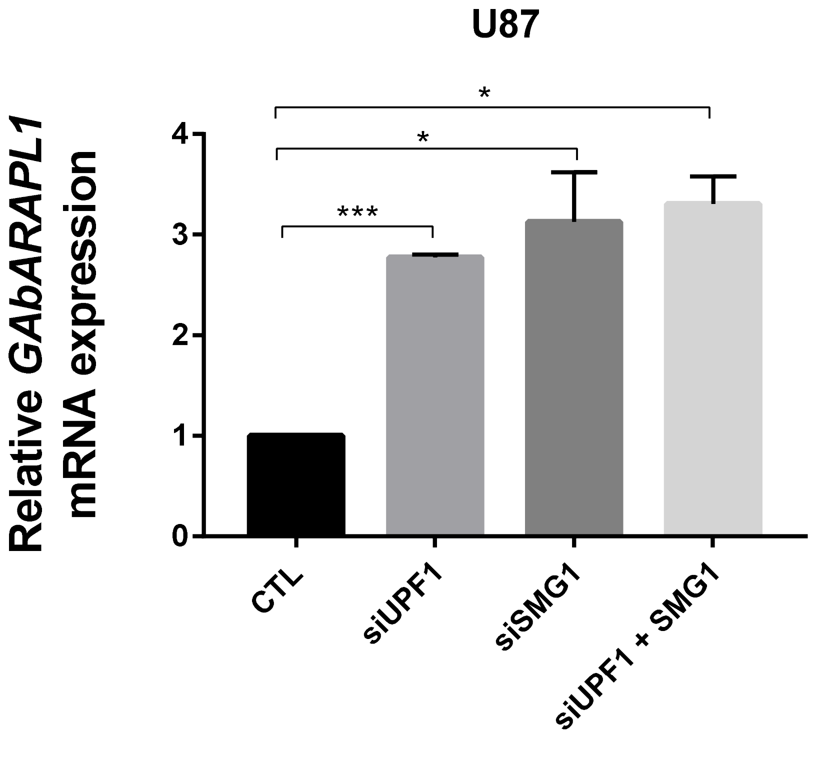

Figure S3. Regulation of the GABARAPL1 mRNA expression upon siRNA-mediated inhibition of the NMD. *U87 cells have been transfected with siRNA (20 pmol/well) targeting NMD transcripts UPF1 or SMG1. Quantification by RT-qPCR of GABARAPL1 transcripts expression levels. The values were calculated using the ΔΔCT method and were normalized to H3B2 levels and expressed as fold changes. Data are represented as ± S.E.M of three independent experiments. P-values were calculated using a Student's t test.*

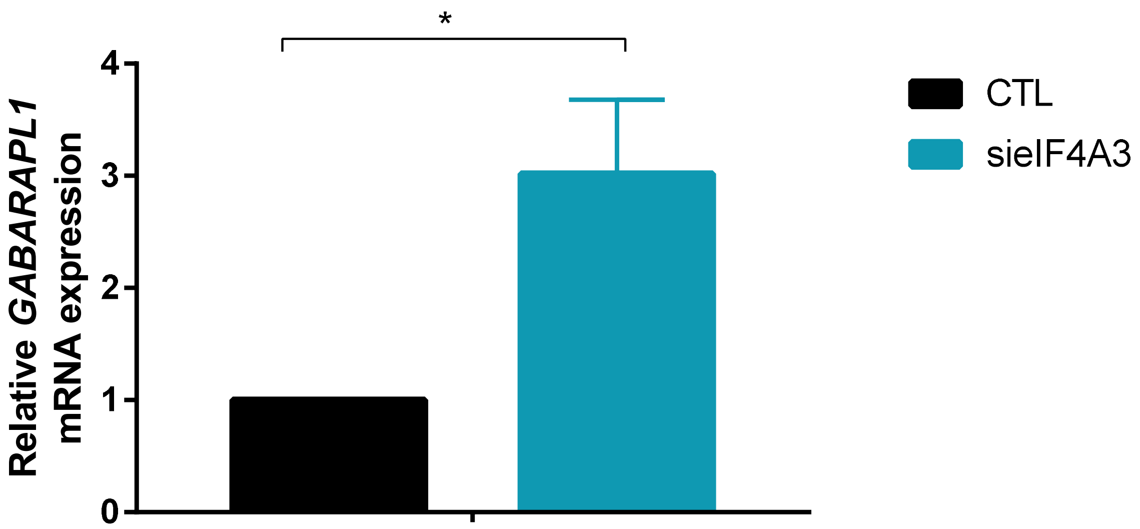

Figure S4. Regulation of the GABARAPL1 mRNA levels upon siRNA-mediated inhibition of eIF4A3. *SiHa cells have been transfected with siRNA (20 pmol/well) targeting eIF4A3. Quantification by RT-qPCR of expression levels of GABARAPL1 transcripts. The values were calculated using the ΔΔCT method and were normalized to H3B2 levels and expressed as fold changes. Data are represented as ±S.E.M of three independent experiments. P-values were calculated using a Student's t test.*
